# The Mechanisms of Human Renal Epithelial Cell Modulation of Autologous Dendritic Cell Phenotype and Function

**DOI:** 10.1371/journal.pone.0134688

**Published:** 2015-07-31

**Authors:** Sandeep Sampangi, Andrew J. Kassianos, Xiangju Wang, Kenneth W. Beagley, Travis Klein, Sadia Afrin, Helen Healy, Ray Wilkinson

**Affiliations:** 1 Conjoint Kidney Research Laboratory, Pathology Queensland, Brisbane, Queensland, Australia; 2 Department of Renal Medicine, Royal Brisbane and Women’s Hospital, Brisbane, Queensland, Australia; 3 Institute of Health and Biomedical Innovation, Queensland University of Technology, Brisbane, Queensland, Australia; 4 Medical School, University of Queensland, Brisbane, Queensland, Australia; UNIFESP Federal University of São Paulo, BRAZIL

## Abstract

Proximal tubule epithelial cells (PTEC) of the kidney line the proximal tubule downstream of the glomerulus and play a major role in the re-absorption of small molecular weight proteins that may pass through the glomerular filtration process. In the perturbed disease state PTEC also contribute to the inflammatory disease process via both positive and negative mechanisms via the production of inflammatory cytokines which chemo-attract leukocytes and the subsequent down-modulation of these cells to prevent uncontrolled inflammatory responses. It is well established that dendritic cells are responsible for the initiation and direction of adaptive immune responses. Both resident and infiltrating dendritic cells are localised within the tubulointerstitium of the renal cortex, in close apposition to PTEC, in inflammatory disease states. We previously demonstrated that inflammatory PTEC are able to modulate autologous human dendritic cell phenotype and functional responses. Here we extend these findings to characterise the mechanisms of this PTEC immune-modulation using primary human PTEC and autologous monocyte-derived dendritic cells (MoDC) as the model system. We demonstrate that PTEC express three inhibitory molecules: (i) cell surface PD-L1 that induces MoDC expression of PD-L1; (ii) intracellular IDO that maintains the expression of MoDC CD14, drives the expression of CD80, PD-L1 and IL-10 by MoDC and inhibits T cell stimulatory capacity; and (iii) soluble HLA-G (sHLA-G) that inhibits HLA-DR and induces IL-10 expression by MoDC. Collectively the results demonstrate that primary human PTEC are able to modulate autologous DC phenotype and function via multiple complex pathways. Further dissection of these pathways is essential to target therapeutic strategies in the treatment of inflammatory kidney disorders.

## Introduction

Proximal tubule epithelial cells (PTEC) of the kidney line the proximal tubule downstream of the glomerulus where one of their major physiological roles is the re-absorption of small molecular weight proteins, peptides and glucose via receptors present on the villi of their luminal surface. This localisation and biological role exposes PTEC to numerous challenging stimuli in the event of any up-stream pathology, with excessive protein, glucose, toxins, advanced glycation end products (AGEs) and bacterial products all being able to perturb normal PTEC physiology [1–3]. In this perturbed state, PTEC are thought to be central players in the disease process, where they alter their gene transcription profiles [4] and secrete a range of chemoattractant cytokines to recruit immune cells into the interstitium of the kidney [5]. In addition to such changes, we have recently published that these PTEC are also able to modulate autologous immune-cell phenotype and function [6–8].

Dendritic cells (DC) are a powerful population in the innate immune system. They are professional antigen presenting cells (APC) that, following activation in the presence of danger signals, induce and regulate adaptive immune responses. Triggering of DC leads to the up-regulation of co-stimulatory (CD80, CD86) and inhibitory (PD-L1) molecules, production of pro-inflammatory (IL-12) and regulatory (IL-10) cytokines and priming of T cell responses [9].

In humans, two subclasses of DC that have been frequently reported within diseased kidney are (i) monocyte-derived DC (MoDC), an inflammatory DC population that rapidly develop from blood monocytes in response to inflammation and infection and (ii) CD1c^+^ DC, a prominent subclass of the myeloid DC lineage [5, 10–12]. In all of these studies, DC were reported as being primarily localised to the interstitium, with minimal to no evidence for their homing to the glomerular compartment. The restricted localisation of infiltrating DC to the interstitium positions them to receive the signals of activated PTEC. Indeed, we have published that human PTEC are able to modulate autologous MoDC and CD1c^+^ DC surface antigen (Ag) expression, Ag-uptake, cytokine expression and T cell stimulatory function. [7].

In this current report we extend these findings to characterise the mechanisms of PTEC modulation of DC phenotype and function using primary human PTEC and autologous MoDC as the model system. We focus, in particular, on cell surface-expressed PD-L1, soluble HLA-G (sHLA-G) and intracellular indoleamine-2,3-dioxygenase (IDO) molecules expressed by primary human PTEC, as we have recently demonstrated their immuno-regulatory effects on autologous T and B lymphocytes [6, 8].

## Materials and methods

### Subjects

Kidney tissue from the healthy portion of malignant and non-malignant nephrectomies and peripheral blood from these same donors three to six months post-nephrectomy were obtained following written informed consent under approval by the Queensland Institute of Medical Research (P293) and Royal Brisbane and Women’s Hospital (2002/011) Ethics Committees.

### Isolation and culture of PTEC

Cortex tissue was dissected from macroscopically/microscopically normal portions of the kidney and processed for PTEC purification within one hour. PTEC were purified following the method of Glynne and Evans [13] and cultured in Defined Medium (DM). DM comprised a 1:1 mixture of Dulbecco’s Modified Eagle’s Medium and Ham’s F12 containing 15mM HEPES buffer, L-glutamine and pyridoxine hydrochloride (Invitrogen, Grand Island, NY, USA). The medium was supplemented with epidermal growth factor (10 ng/ml), insulin (10 μg/ml), transferrin (5 μg/ml), selenium (5 ng/ml), hydrocortisone (36 ng/ml), triiodothyronine (4 pg/ml), penicillin (50 U/ml) and streptomycin (50 mg/ml). Cell stocks were frozen at passage 1 (P1) and all PTEC used in experiments were at P2 or P3. PTEC were characterised on the basis of: 1) strong staining for cytokeratin-18, 2) strong staining for alkaline phosphatase activity using the naphtal AS-MX method and 3) characteristic cobblestone morphology.

### Activation of PTEC

PTEC were cultured in DM until 70–80% confluence. As previously established [7], PTEC were then exposed to 100ng/ml interferon (IFN)-γ (R&D Systems, Minneapolis, MN, USA) for 24 hours to recapitulate the inflammatory disease setting. To prevent further proliferation, PTEC were irradiated with 3000cGy prior to co-culture with monocytes.

### MoDC generation and culture

Peripheral blood mononuclear cells (PBMC) were isolated using Ficoll-Paque Plus density gradient centrifugation (GE Healthcare, Uppsala, Sweden). Monocytes were isolated from PBMC by CD14^+^ immuno-magnetic selection (>95% purity) (Mitenyi Biotec, Bergisch Gladbach, Germany) according to the manufacturer’s instructions. For differentiation into MoDC, purified monocytes were cultured for 5 days in Complete Medium (CM) consisting of RPMI 1640, containing 10% heat-inactivated human AB serum, 100U/ml penicillin, 100μg/ml streptomycin, 2mM L-glutamine, 1mM sodium pyruvate, 0.1mM non-essential amino acids, 10mM HEPES buffer solution (all from Invitrogen), and 50μM 2-mercaptoethanol (Sigma-Aldrich, St Louis, MO, USA) and supplemented with 800U/ml GM-CSF (Miltenyi Biotec) and 1000U/ml IL-4 (Miltenyi Biotec). Cultures were established in either; standard 24-well plates for contact-dependant (CD) cultures or 0.4μM transwell 24 well plates for contact-independent (CI) cultures, alone (Ctrl-MoDC) or in the presence of IFN-γ-activated, irradiated PTEC (PTEC-MoDC).

### Blocking studies

For programmed death ligand-1 (PD-L1) blocking, 10 μg/mL of anti-human PD-L1 (Biolegend, clone 29E.2A3, San Diego, CA, USA) blocking antibody was added to the surface of PTEC and incubated for 2 h. Following incubation, the PTEC wells were extensively washed with 5 changes of warm CM to remove all traces of unbound antibody and the co-cultures were established. To neutralise soluble human leukocyte antigen-G (sHLA-G) and IDO, 10 μg/mL of anti-human sHLA-G (Biolegend, clone 87G) blocking antibody and 1500 μM/mL IDO inhibitor 1-methyl-tryptophan (1-MT) (Life Technologies, CA, USA) respectively was added into the PTEC or control wells immediately following co-culture establishment. The effective blocking concentration levels of 1-MT were determined via titration using cell viability and tryptophan-kynurenine ratios monitored by HPLC (data not shown).

### Flow cytometry phenotyping

Cells were labelled with combinations of PE-, FITC-, PerCP-, APC-, PE-Cy7-, V450- and Pacific Orange-conjugated mouse anti-human CD14, CD80, CD86, DC-SIGN (CD209) (all from BD Biosciences, Franklin Lakes, NJ, USA), PD-L1 (CD274) (Biolegend) and HLA-DR (Invitrogen) antibodies and appropriate isotype controls, using 0.25 μg of each antibody per staining reaction. Acquisition was performed on a BD FACSCanto II flow cytometer (BD Biosciences) and analysis of flow data was performed using FlowJo 7.6.4 (Tree Star, Inc., Ashland, OR, USA). For viability staining, cells were labelled with a Near-IR dead cell stain kit (Invitrogen) according to the manufacturer’s instructions and analysed by flow cytometry.

### Cytokine secretion assays

Culture supernatants were harvested and levels of IL-10 and IL-12p70 were determined using Flow Cytometric Bead Arrays (BD Biosciences) according to the manufacturer’s instructions.

### Allogeneic MLR

Allogeneic CD4^+^ T cells were isolated from PBMC by negative immuno-magnetic selection using the CD4^+^ T cell isolation kit II (>95% purity) (Miltenyi Biotec). MoDC separated from PTEC, were plated in triplicate in 96-well plates at 1:2 serial dilutions starting at 2.5x10^4^ cells/well. Allogeneic CD4^+^ T cell responders were added at 1x10^5^ cells/well. Cells were cultured for 5 days and proliferation was assessed by the addition of 1μCi ^3^H-thymidine/well (Perkin Elmer, Boston, MA, USA) for the last 8 hours of culture.

### Statistical Analysis

Comparisons between two groups were performed using a two-tailed Wilcoxon signed rank test. Statistical tests were performed using GraphPad Prism 5.0 analysis software (GraphPad, San Diego, CA, USA). P values ≤0.05 were considered statistically significant.

## Results

We first undertook contact-dependent and transwell contact-independent parallel co-culture experiments to establish whether PTEC modulation of autologous DC phenotype and function operated through contact or soluble mechanisms.

### Autologous PTEC modulate the MoDC expression of HLA-DR, CD86 and CD80 by contact independent mechanisms and CD14 and PD-L1 by both contact dependent and contact independent mechanisms

**Fig 1:**
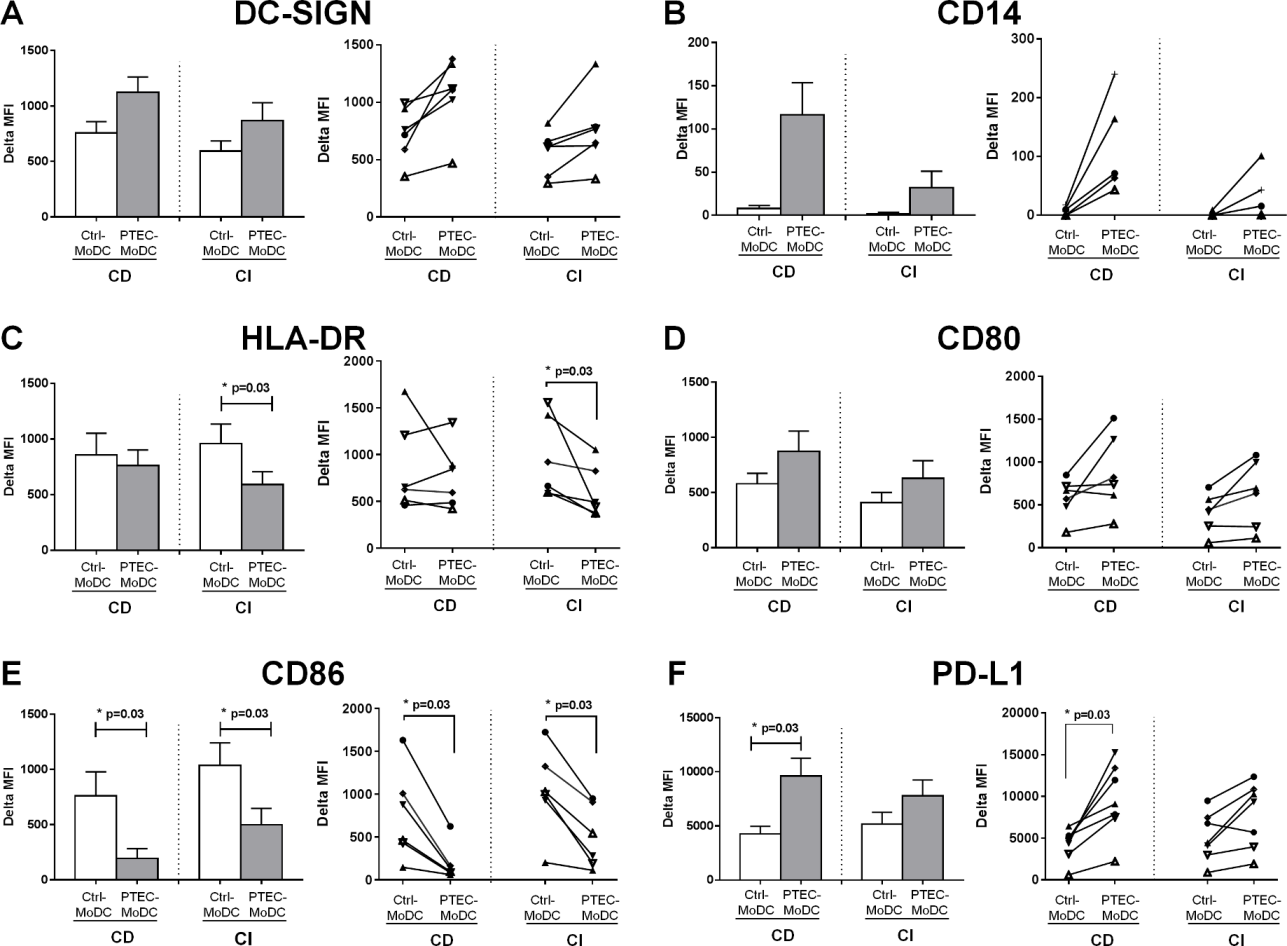
Expression of surface antigens (A) DC-SIGN, (B) CD14, (C) HLA-DR, (D) CD80, (E) CD86 and (F) PD-L1 by Ctrl- and PTEC-MoDC from CD and CI culture systems. The bar graph represents mean values (± SEM) for five to seven donors and the line graph represents five to seven individual donor experiments. Surface expression was measured by flow cytometry (gated on live, single cells) and reported as delta MFI (MFI test-MFI isotype control). Statistical comparisons between the two groups were performed using a two-tailed Wilcoxon signed rank test.

In both CD and CI culture systems the progression of CD14^+^ monocyte differentiation into MoDC was confirmed by the expression of the DC marker DC-SIGN (Fig 1A) in both the absence and presence of PTEC. The expression of CD14 was retained at elevated levels by PTEC-MoDC when compared with Ctrl-MoDC from both CD and CI culture systems but the level of CD14 was considerably less on PTEC-MoDC in the CI culture system when compared with PTEC-MoDC from the CD culture system (Fig 1B). The expression of HLA-DR was down regulated by PTEC-MoDC in three out of six donors in the CD culture system and in all donors from the CI culture system (Fig 1C). The expression of CD86 on PTEC-MoDC was significantly down regulated in all donors in both CD and CI culture systems (Fig 1E) and CD80 was upregulated by PTEC-MoDC in four out of six donors in the CD system and in five out of six donors in the CI system, when compared with Ctrl-MoDC (Fig 1D). The expression of PD-L1 was up regulated on PTEC-MoDC in both CD and CI culture systems, but this up-regulation was only significant (p = 0.03) in the CD culture system (Fig 1F).

### Autologous PTEC modulate IL-10 secretion by MoDC through CD mechanisms

**Fig 2:**
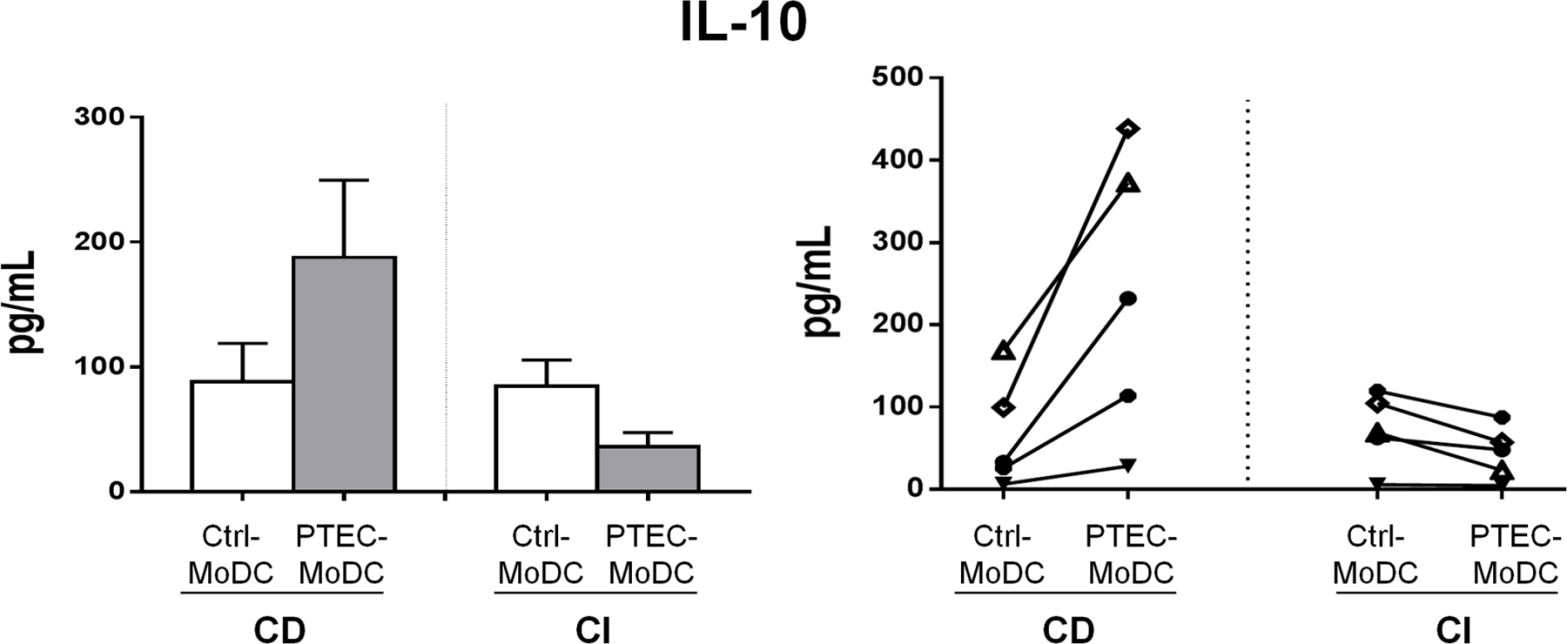
Secretion of anti-inflammatory cytokine IL-10 by Ctrl- and PTEC-MoDC from CD and CI culture systems represented as mean values for five donors (bar graph) and five individual donor (line graph) experiments as determined using a flow cytometric bead array and presented as pg/mL.

To identify the regulatory mechanism of autologous PTEC on MoDC cytokine secretion, we analysed the supernatant of Ctrl- and PTEC-MoDC from both CD and CI culture systems. PTEC-MoDC secreted elevated levels of IL-10 in all five donors when compared with Ctrl-MoDC within the CD culture system. However, this increased IL-10 expression was not observed in the CI culture system (Fig 2). Secretion of pro-inflammatory cytokine IL-12p70 was not detec in any culture system (data not shown).

### PTEC-MoDC from both CD and CI culture systems were less effective in stimulating allogeneic CD4^+^ T cell responses

**Fig 3:**
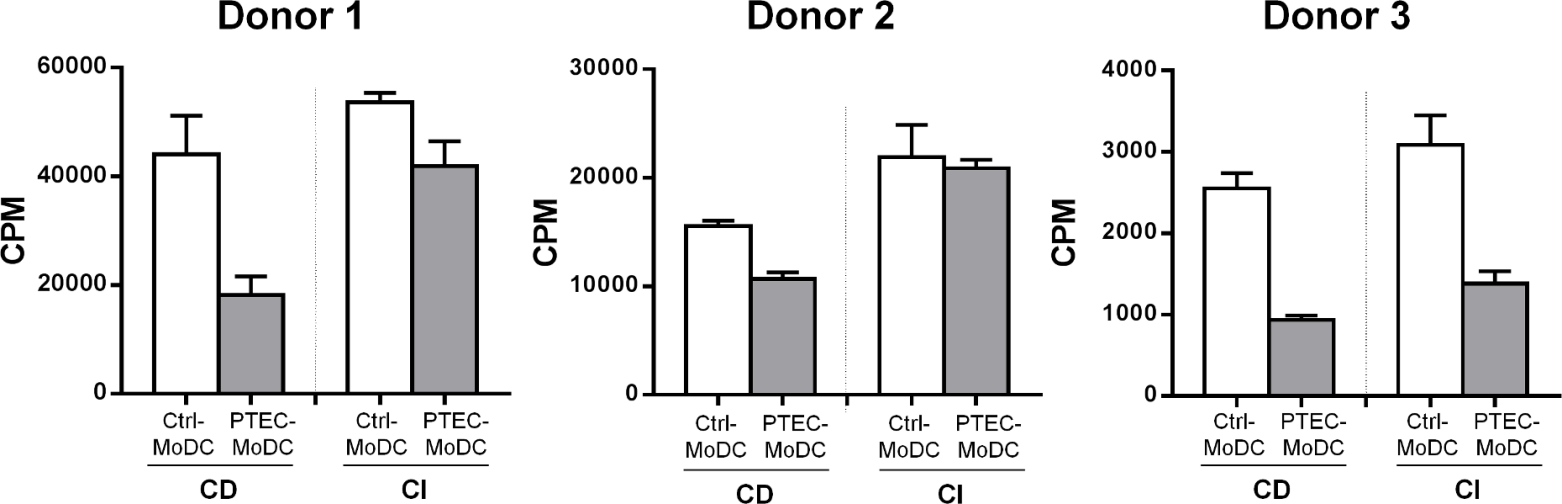
Allogeneic CD4^+^ T cell proliferation induced by Ctrl- and PTEC-MoDC from CD and CI cultures in three individual donors (donor 1, donor 2 and donor 3) at 1:8 MoDC:T cell ratio. Proliferation was measured by ^3^[H]-thymidine incorporation and expressed as counts per minute (CPM) after 5 days.

The ability to induce proliferation of allogeneic CD4^+^ T cells in a mixed lymphocyte reaction (MLR) is one of the definitive features of DC. To identify the mechanism through which autologous PTEC modulate MoDC to down-regulate allogeneic CD4^+^ T cell responses, allo-MLR assays were established utilising Ctrl- and PTEC-MoDC obtained from CD and CI culture systems. PTEC-MoDC were less effective at stimulating CD4+ T cell responses when compared with their respective Ctrl-MoDC from both CD and CI culture systems. Although PTEC-MoDC from CI cultures stimulated higher levels of T cell proliferation compared to PTEC from CD co-cultures, suggesting a partially contact-mediated mechanism for PTEC suppression (Fig 3), this failed to reach significance.

Following the elucidation of both CD and CI mechanisms of immune regulation, the molecules participating in these mechanisms were investigated. As we have identified that PTEC express the immuno-regulatory molecules sHLA-G, PD-L1 and IDO [8], the specific role of these molecules in modulating MoDC phenotype and function was investigated by blocking their activity within the PTEC-MoDC co-cultures.

### PD-L1 expressed by autologous PTEC mediates the expression of PD-L1 on MoDC but does not impact IL-10 secretion by these cells

**Fig 4:**
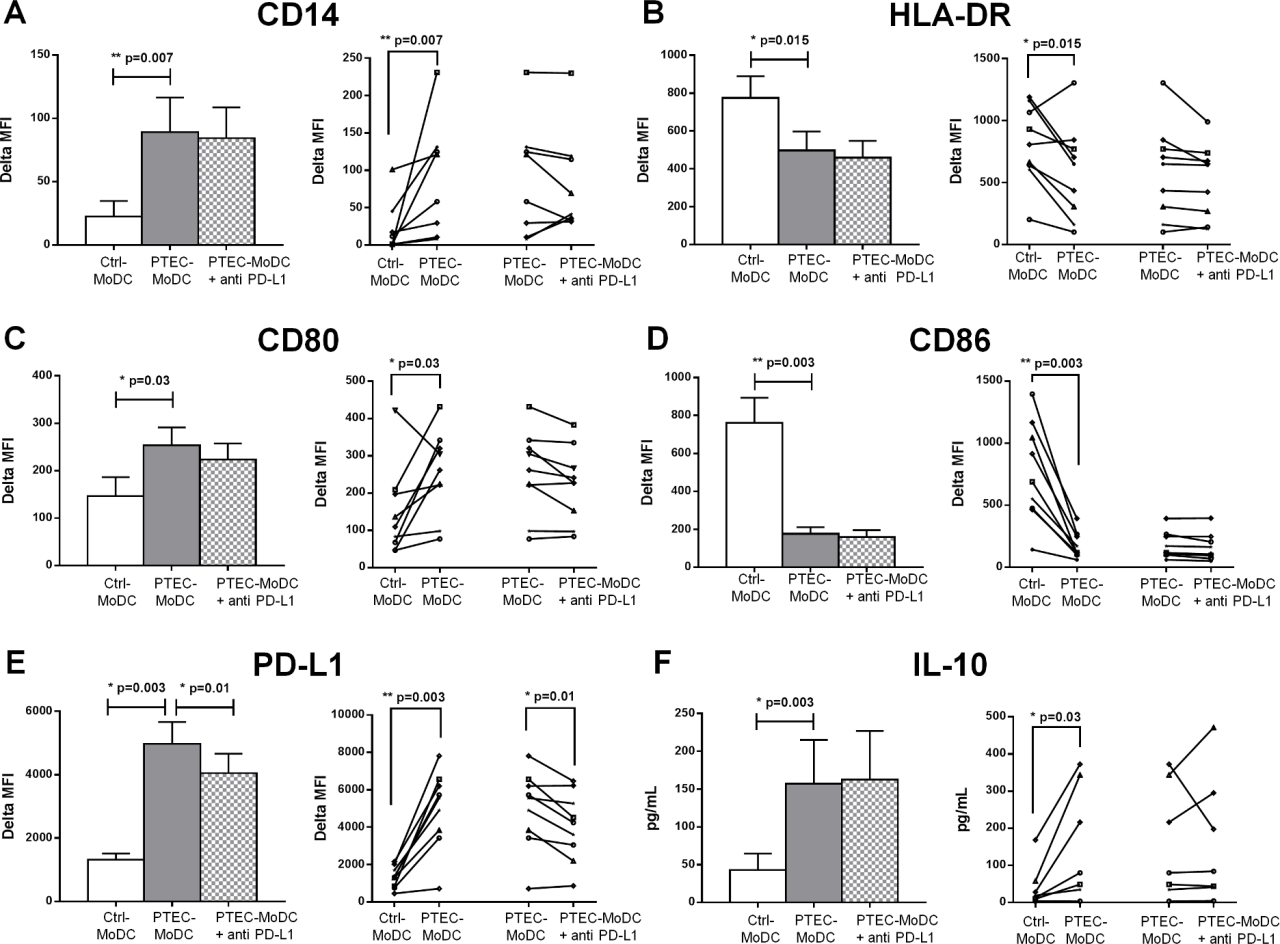
Expression of surface antigens (A) CD14, (B) HLA-DR, (C) CD80, (D) CD86, (E) PD-L1 and (F) anti-inflammatory cytokine IL-10 by Ctrl-MoDC, PTEC-MoDC and PTEC-MoDC supplemented with blocking antibody for PD-L1. The bar graph represents mean of eight to nine donors and the line graph represents individual donor experiments. Surface expression was measured by flow cytometry (gated on live, single cells) and reported as delta MFI (MFI test-MFI isotype control). Cytokine expression was determined using a flow cytometric bead array and presented as pg/mL.

PTEC PD-L1 blocking significantly decreased (p = 0.01) PD-L1 expression on PTEC-MoDC (Fig 4E). The expression of other surface antigens including DC-SIGN (data not shown), HLA-DR (Fig 4B), CD80 (Fig 4C) and CD86 (Fig 4D) were not influenced by PTEC PD-L1 blocking. Similarly, levels of IL-10 were unaffected by PTEC PD-L1 blocking (Fig 4F) indicating that PTEC expressed PD-L1 was not involved in the regulation of observed elevated cytokine expression levels.

### sHLA-G expressed by autologous PTEC partly mediates the expression of HLA-DR on MoDC and the secretion of IL-10 by MoDC

**Fig 5:**
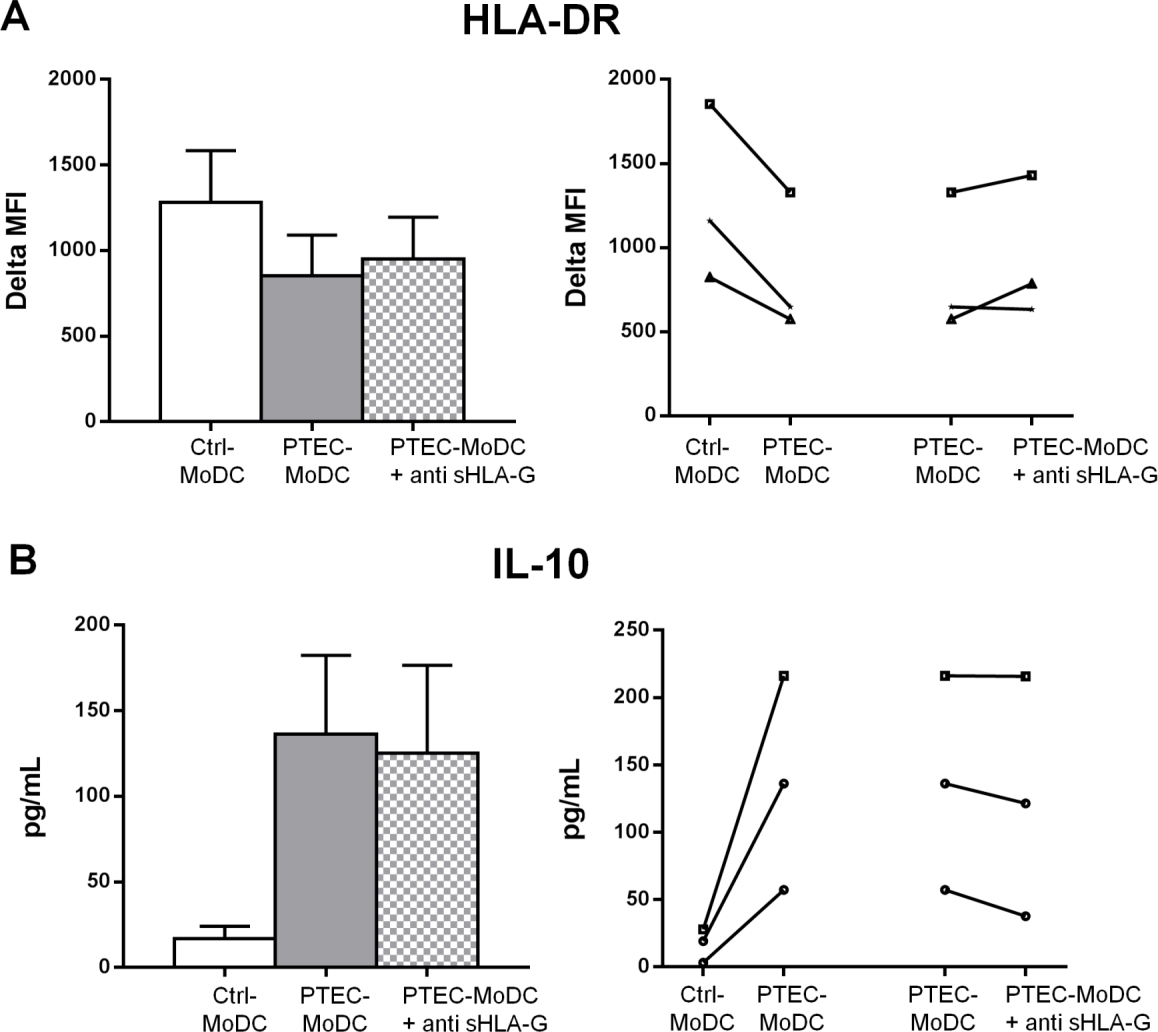
Expression of surface antigen (A) HLA-DR and (B) anti-inflammatory cytokine IL-10 by Ctrl-MoDC, PTEC-MoDC and PTEC-MoDC supplemented with blocking antibody for sHLA-G. The bar graph represents mean of three donors and the line graph represents individual donor experiments. Surface expression was measured by flow cytometry (gated on live, single cells) and reported as delta MFI (MFI test-MFI isotype control). Cytokine expression was determined using a flow cytometric bead array and presented as pg/mL

Following differentiation, the Ctrl- and PTEC-MoDC were harvested and stained for surface antigen expression and the culture supernatants analysed for IL-10 levels. The blocking of sHLA-G reversed HLA-DR expression on PTEC-MoDC, where two out of three donors demonstrated a partial restoration in the expression of this molecule (Fig 5A). The expression of CD14, CD80, CD86 and PD-L1 were unaffected by sHLA-G blocking (data not shown).

The levels of IL-10 were partly reduced in all donors in the presence of blocking antibody to sHLA-G (Fig 5B). This result was somewhat surprising given that our CD/CI experiments indicated a CD mechanism was responsible for the elevated IL-10 from MoDC in the presence of INF-γ activated autologous PTEC.

### IDO activity of autologous PTEC regulates the expression of CD14, CD80 and PD-L1 on MoDC and partially regulates the secretion of IL-10 from these cells

**Fig 6:**
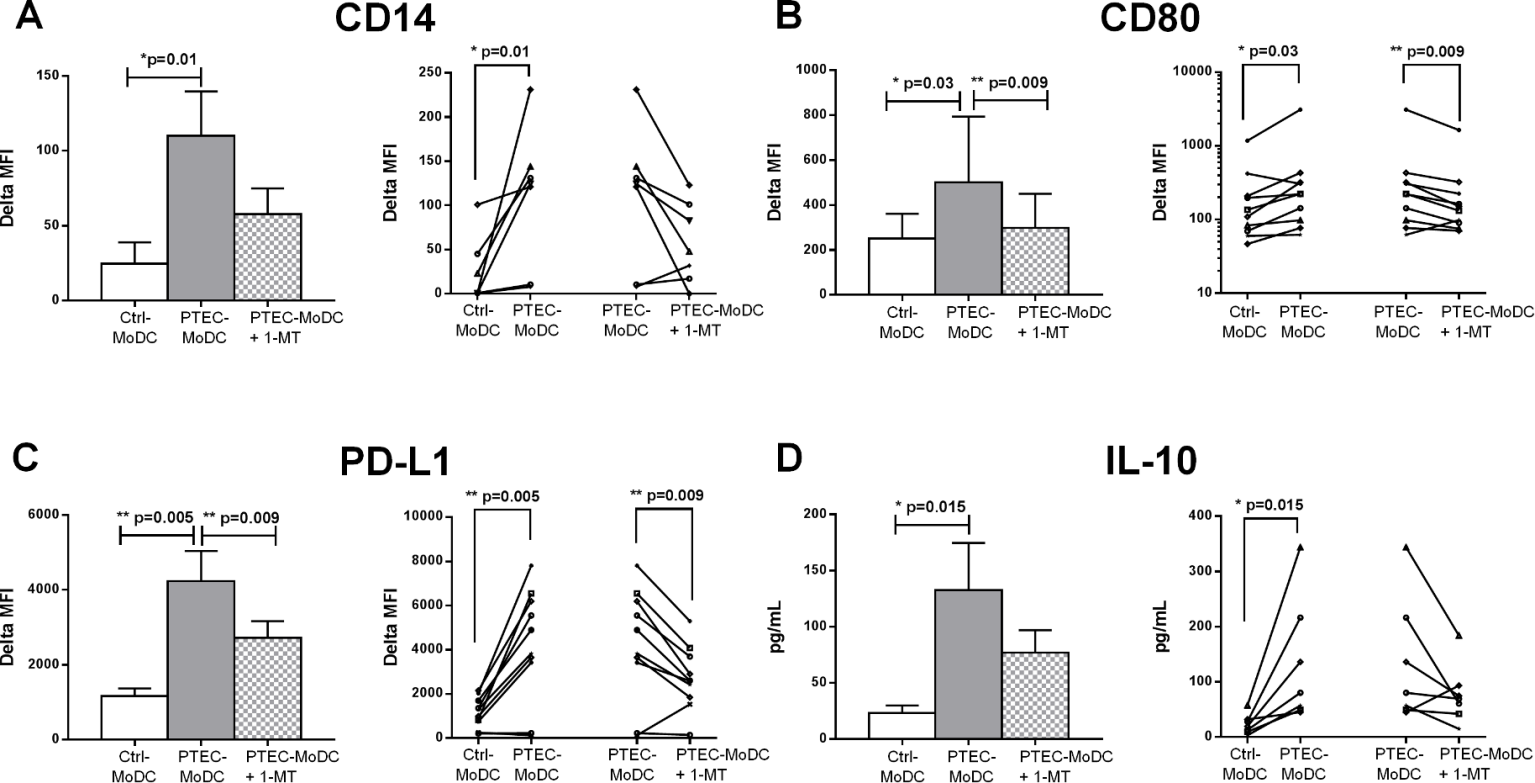
Expression of surface antigens (A) CD14, (B) CD80, (C) PD-L1 and (F) anti-inflammatory cytokine IL-10 by Ctrl-MoDC, PTEC-MoDC and PTEC-MoDC supplemented with 1-MT, an inhibitory molecule to suppress the activity of IDO expressed by autologous PTEC. The bar graph represents mean of seven to ten donors and the line graph represents individual donor experiments. Surface expression was measured by flow cytometry (gated on live, single cells) and reported as delta MFI (MFI test-MFI isotype control). Cytokine expression was determined using a flow cytometric bead array and presented as pg/mL.

Blocking IDO activity in our co-cultures decreased the expression of CD14 in five out of seven donors (Fig 6A), suggesting greater MoDC differentiation of these cells, and significantly down-regulated the expression of CD80 and PD-L1 (Fig 6B and 6C), whilst having no effect on HLA-DR or CD86 levels (data not shown). Blocking IDO activity in our co-cultures also partly down-regulated the expression of IL-10 by PTEC-MoDC in six out of seven donors (Fig 6D).

### IDO activity of autologous PTEC plays a partial role in regulating the allogeneic response-inducing function of MoDC

**Fig 7:**
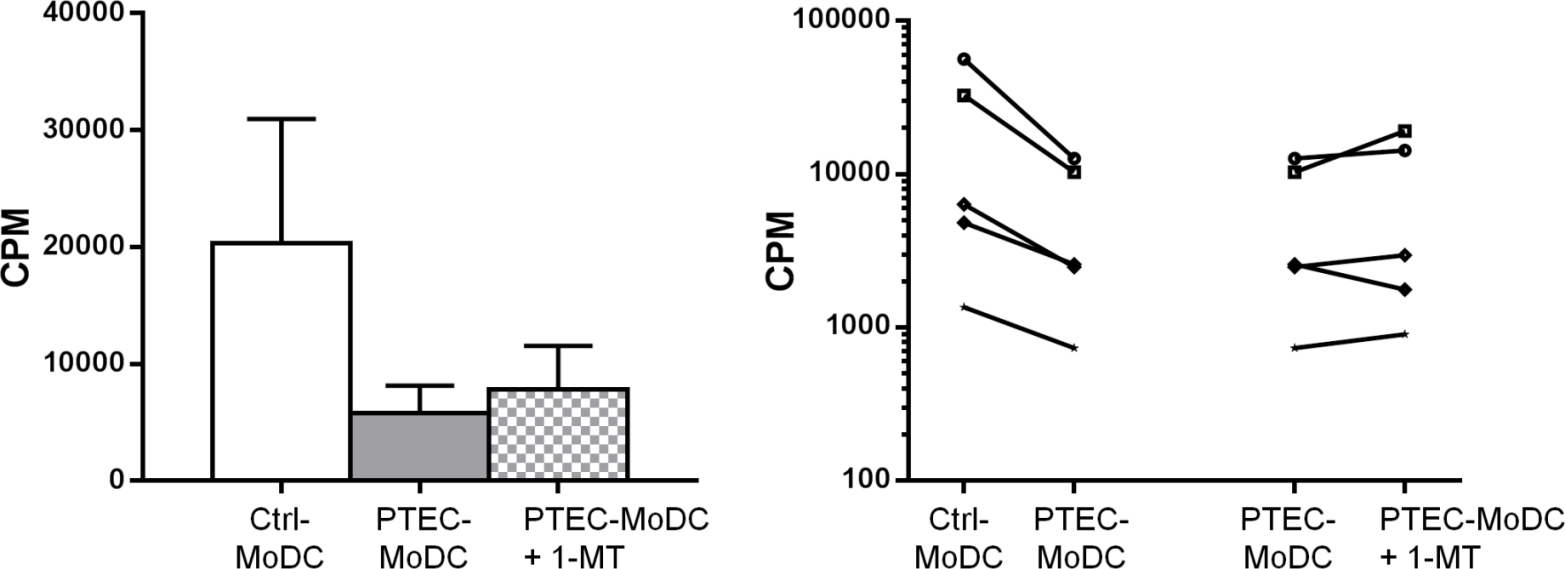
Allogeneic CD4^+^ T cell proliferation induced by Ctrl-MoDC, PTEC-MoDC and PTEC-MoDC supplemented with IDO inhibitor 1-MT represented as mean values for five donors (bar graph) and five individual donors (line graph) at a 1:8 MoDC:T cell ratio. Proliferation was measured by ^3^[H]-thymidine incorporation and expressed as counts per minute (CPM) after 5 days.

To investigate the functional effects of PTEC inhibitory molecules, we established allogeneic MLR with PTEC-MoDC that were differentiated in the presence of anti-PD-L1, anti-sHLA-G and 1-MT respectively. No recovery of allo-stimulatory ability was seen from PTEC-MoDC that were differentiated in the presence of anti-PD-L1 or anti-sHLA-G (data not shown). However, we observed partial restoration of CD4^+^ T cell proliferation in four out of five donors (Fig 7) when PTEC-MoDC were differentiated in the presence of 1-MT, although this restoration did not achieve significance.

## Discussion

As we have previously published [7], the presence of autologous PTEC skew MoDC to become phenotypically less mature and functionally less stimulatory. In this report we investigated the mechanisms by which these regulatory steps occur. As an initial step in this process, CD14^+^ monocytes were differentiated into MoDC in CD or CI culture conditions to identify whether autologous PTEC regulate MoDC differentiation and function by surface expressed molecules (CD mechanism) or by soluble factors (CI mechanism).

Confirming our previous findings [7], all MoDC differentiated in the presence of autologous PTEC in CD culture systems phenotypically retained the monocyte marker CD14, expressed low levels of HLA-DR and CD86 and up-regulated PD-L1. Although the intensity of the expression of CD14, CD80, CD86, HLA-DR, and PD-L1 on Ctrl- and PTEC-MoDC varied between CD and CI culture systems, the overall pattern of expression of these molecules suggested that autologous PTEC regulated CD80, CD86 and HLA-DR by CI mechanisms and CD14 and PD-L1 through both CD and CI mechanisms.

The mechanism/s of autologous PTEC regulation of MoDC function, including their cytokine profiles and their ability to stimulate allogeneic CD4^+^ T cell responses were also investigated by using Ctrl- and PTEC-MoDC derived from both CD and CI culture systems. Autologous PTEC up-regulated the expression of IL-10 from MoDC through a CD mechanism. The allo-MLR assay showed that PTEC-MoDC from both CD and CI culture systems were less effective at stimulating allogeneic CD4^+^ T cell proliferation than Ctrl-MoDC. However, a partial restoration in T cell stimulation by PTEC-MoDC from CI cultures compared to PTEC-MoDC from CD cultures suggests that autologous PTEC inhibit the allogeneic-inducing function of MoDC through both CD and CI mechanisms.

Following the identification of CD or CI regulatory mechanisms behind the expression of phenotypic markers and function of MoDC, molecules participating in these mechanisms were investigated. The up-regulation of immuno-regulatory molecule PD-L1 on autologous PTEC was reported in our previous publication [6]. PD-L1 or CD274, is a inhibitory molecule of the B7 family group which interacts with its receptor PD-1 on monocytes [14], to provide negative signals via the PD-1 cytoplasmic immuno-receptor tyrosine based inhibitory motif (ITIM) [15]. PD-L1 is expressed at low to negligible levels in normal kidney but is up-regulated in a number of kidney diseases, especially within areas of mononuclear cell infiltration [16, 17], suggesting that cytokines secreted from these infiltrating cells may induce PD-L1 expression on PTEC *in vivo*. Our blocking studies indicate that a pivotal role of PTEC-expressed PD-L1 is to increase the expression of PD-L1 on autologous MoDC. As PD-L1 is surface expressed, it was logical to conclude that this was one of the primary CD mechanisms responsible for our observed low T cell stimulatory capacity of PTEC-MoDC. However, our functional PD-L1 blocking studies did not support this assumption.

Our subsequent functional blocking experiments identified IDO as the candidate molecule responsible for this mechanism. The IFN-γ-inducible intracellular enzyme IDO catalyses the breakdown of essential amino acid tryptophan into kynurenine and creates a micro-environment devoid of tryptophan. Tryptophan depletion and the generated kynurenine bi-products have both been recognised as immune-regulators of antigen presenting cells [18–21]. We have recently identified that our IFN-γ treated primary PTEC cultures express biologically active IDO [8]. IDO expression has been demonstrated within the kidney compartment and increased levels have correlated with improved outcomes in several mouse models of human kidney disease [22], suggesting this molecule may play a role in dampening inflammatory responses. However, Mohib et al demonstrated a pathological role for IDO in ischemia-reperfusion injury [23].

Our results contribute to this apparent dichotomy by demonstrating that PTEC-produced IDO both up-regulates costimulatory CD80 and inhibitory PD-L1 and regulatory IL-10 on APC. In addition, our results show that PTEC-expressed IDO plays a role in the retention of monocyte marker CD14 on developing MoDC, inhibiting complete monocyte-to-MoDC differentiation and contributing to our observed weak T cell stimulatory capacity of these cells. However, it should be noted from our transwell experiments that IL-10 expression from developing MoDC is contact-dependant, indicating that the soluble effects of tryptophan depletion upon IL-10 induction could be driving an autocrine up-regulation of another, as yet un-identified, immuno-regulatory surface molecule on PTEC.

Our results also implicated another soluble inhibitory molecule, sHLA-G, in the decreased expression of HLA-DR and induction of IL-10 from MoDC. We have demonstrated HLA-G mRNA and intracellular protein up-regulation in our primary PTEC cultures in response to IFN-γ stimulation (data not shown), with concomitant increases in sHLA-G from the supernatants of these cells [8]. This is in contrast to a previous study by Kronsteiner et al [24] which reported that PTEC alone do not secrete sHLA-G. However, unlike our study, Kronsteiner *et al* did not stimulate PTEC with IFN-γ, which induces the expression of sHLA-G. Functionally, the role of sHLA-G has been demonstrated to be beneficial in various clinical conditions including pregnancy, transplantation and various inflammatory diseases [25, 26]. In kidney models, the presence of HLA-G in renal allograft biopsies [27] and increased sHLA-G levels in plasma samples from kidney transplant patients [28] have both correlated with improved kidney graft acceptance. Our observations that PTEC-expressed sHLA-G is partially responsible for the low HLA-DR and increased IL-10 by our PTEC-MoDC may provide a mechanistic answer to the findings in those studies.

Collectively, our results confirm that primary human PTEC are able to modulate autologous APC phenotype and function via multiple complex interactive pathways including PTEC-PD-L1 induction of MoDC PD-L1; IDO maintenance of CD14 and induction of CD80, PD-L1 and IL-10 expression on MoDC; and sHLA-G inhibition of HLA-DR up-regulation and induction of IL-10 expression from MoDC. *In–vivo* these pathways may be cumulative, with the net outcome on DC functional response dependent upon multiple factors including the stage of the disease process, functional competency of the resident PTEC, the cytokine milieu present within the interstitium and the developmental/activation stage of responding DC. Overall however, we believe these mechanisms have evolved to enable PTEC to act as negative regulatory cells to down-modulate DC function within the early inflammatory setting and to control unrestrained inflammation.

Further dissection of these pathways should enable the development of novel immunotherapy strategies for the treatment of inflammatory chronic kidney disease.
